# Features of a Mobile Support App for Patients With Chronic Obstructive Pulmonary Disease: Literature Review and Current Applications

**DOI:** 10.2196/mhealth.4951

**Published:** 2017-02-20

**Authors:** Drishty D Sobnath, Nada Philip, Reem Kayyali, Shereen Nabhani-Gebara, Barbara Pierscionek, Anouk W Vaes, Martijn A Spruit, Evangelos Kaimakamis

**Affiliations:** ^1^ Digital Media for Health, Medical Information and Network Technology Faculty of Science, Engineering and Computing Kingston University London Surrey United Kingdom; ^2^ CIRO Research and Education Horn Netherlands; ^3^ Pulmonary Clinic George Papanikolaou General Hospital Thessaloniki Greece

**Keywords:** mHealth, COPD app, COPD support tool, self-management application, chronic diseases, application design, WELCOME

## Abstract

**Background:**

Chronic obstructive pulmonary disease (COPD) is a serious long-term lung disease in which the airflow from the lungs is progressively reduced. By 2030, COPD will become the third cause of mortality and seventh cause of morbidity worldwide. With advances in technology and mobile communications, significant progress in the mobile health (mHealth) sector has been recently observed. Mobile phones with app capabilities (smartphones) are now considered as potential media for the self-management of certain types of diseases such as asthma, cancer, COPD, or cardiovascular diseases. While many mobile apps for patients with COPD are currently found on the market, there is little published material on the effectiveness of most of them, their features, and their adoption in health care settings.

**Objectives:**

The aim of this study was to search the literature for current systems related to COPD and identify any missing links and studies that were carried out to evaluate the effectiveness of COPD mobile apps. In addition, we reviewed existing mHealth apps from different stores in order to identify features that can be considered in the initial design of a COPD support tool to improve health care services and patient outcomes.

**Methods:**

In total, 206 articles related to COPD management systems were identified from different databases. Irrelevant materials and duplicates were excluded. Of those, 38 articles were reviewed to extract important features. We identified 214 apps from online stores. Following exclusion of irrelevant apps, 48 were selected and 20 of them were downloaded to review some of their common features.

**Results:**

Our review found that out of the 20 apps downloaded, 13 (65%, 13/20) had an education section, 5 (25%, 5/20) consisted of medication and guidelines, 6 (30%, 6/20) included a calendar or diary and other features such as reminders or symptom tracking. There was little published material on the effectiveness of the identified COPD apps. Features such as (1) a social networking tool; (2) personalized education; (3) feedback; (4) e-coaching; and (5) psychological motivation to enhance behavioral change were found to be missing in many of the downloaded apps.

**Conclusions:**

This paper summarizes the features of a COPD patient-support mobile app that can be taken into consideration for the initial design of an integrated care system to encourage the self-management of their condition at home.

## Introduction

Chronic obstructive pulmonary disease (COPD) is a serious long-term lung disease in which the flow of air out of the lungs is progressively reduced. The deterioration in lung function is caused by airway remodeling and progressive loss of lung tissue and damage to the lung parenchyma caused mainly by cigarette smoking. The burden of COPD is huge and is still growing. A World Health Organization (WHO) report anticipates that by 2030, COPD will become the third cause of mortality and seventh cause of morbidity worldwide [1]. It is also a disease state often associated with several comorbidities such as cardiovascular disease, metabolic syndrome, osteoporosis, mental health diseases, and lung cancer [2]. Research has shown that the early, accurate diagnosis of COPD and lifestyle management of patients with COPD can have a crucial impact on handling the long-term condition [3,4]. The treatment of stable COPD is highly dependent on the patient’s symptoms and clinical manifestations.

Today, mobile health (mHealth) is extensively used for health services and patient education. mHealth is a term used to describe the practice of medicine with the support of mobile computing and mobile devices such as tablets, mobile phones, and personal digital assistants (PDAs) for health care. Software apps, specifically designed for and available on mobile devices, have been actively adopted by users of mobile phones and tablets [5]. As of June 2016, according to Apple, 2 million apps were available to download in the Apple Store while the Android Market was offering 2.2 million apps [6]. Mobile apps and bespoke software tools can be used to help people self-manage their health and wellness with convenience while being on the move [7]. Working in the health care system involves extensive mobility of health care professionals as well as communication and collaboration among colleagues and patients. Indeed, the UK Department of Health has recommended that apps be “prescribed” as part of the care for long-term conditions [8]. However, there are few published studies addressing which specific features of mobile health apps offer the greatest potential to benefit patients with COPD in an effective way.

The management of COPD requires a multidisciplinary approach, involving both pharmacological and non-pharmacological treatment. Finding the right features to be incorporated in a support tool for patients with COPD seems to be very challenging. However, some studies show that effective management of COPD through integrated care systems, mHealth, and other technologies has the potential to both benefit the patient and reduce hospitalization costs in long-term management of COPD [9,10]. Comorbidities such as heart failure and diabetes add to the disease burden [11,12]. Providing the patient with the right care at the right time is crucial in order to prevent exacerbations, reduce hospitalization, and reduce mortality risks. Other factors, such as adopting a healthy lifestyle (good nutrition and exercise), result in a better quality of life in patients with COPD [13].

The main objective of this study was to review mobile apps, COPD management systems, and the literature in order to identify features for a COPD mobile support app. The features identified from the literature and from the apps can then be considered in the initial design of an integrated care system for the WELCOME European Union project to fill the missing links of a COPD support tool [14]. The main target user of such a support tool will be mainly patients with COPD including those suffering from different comorbidities. The support tool designed for patients can also be used to record important data on a main database to help health care professionals follow-up on their patients’ health conditions closely.

## Methods

### Pilot Studies and Articles

**Figure 1:**
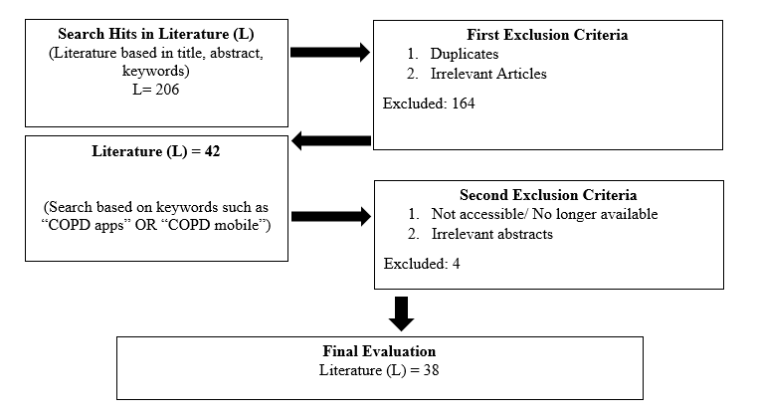
Selection criteria for published articles.

A search was conducted using the term “COPD apps”/“COPD mobile” to retrieve any pilot studies carried out on different health systems or mobile apps for COPD. Many studies retrieved were about prototypes or apps not yet published in any online market. A thorough search was done retrieving data from different databases such as ACM, Science Direct, IEEE Xplore, Medline, Scopus, and Google Scholar from 2009 to 2014. The logical operators “OR” and “AND” were used to identify duplicates. The search was based on the metadata (eg, title, abstract, and keywords). The detailed selection process is shown in Figure 1. A total of 38 articles were downloaded based on the keywords and were reviewed to extract features that could be considered for a COPD support tool.

### Mobile Apps

This first step was carried out by searching the COPD-related apps from app stores such as Google Play Store, iTunes, BlackBerry World, Windows Phone, and Nokia's Ovi store. The European Directory of Health Apps and the National Health Service (NHS) App stores automatically have their apps listed in either Google Play or the Apple Store. A total of 214 apps were identified on the various mobile markets using the search tem “COPD” and “COPD Management.” The Google Android market had the highest results (63.6%, 136/214), followed by the Apple iOS (33.6%, 72/214) and Windows mobile (2.8%, 6/214) platforms. No results were found on the Blackberry store.

**Figure 2:**
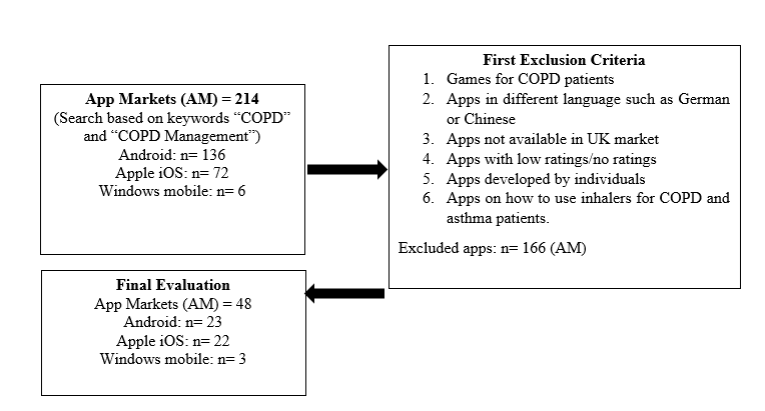
Breakdown of the online markets search process.

A further evaluation was then performed to select relevant apps for this study. Only English-language mHealth apps available in the UK market were selected following the original systematic search. Several criteria had to be considered while selecting apps from their respective markets. The inclusion and exclusion criteria that were considered for this study are shown in Figure 2. The comprehensiveness and consistency of information were assessed for apps presenting health information about COPD. Company-designed apps rather than individual ones were selected. This is to ensure that the apps have been reviewed and have professional recognition. All identified apps are available on the UK market and contain information written in English.

## Results

### Features Identified in Studies

One important feature for an efficient support tool is the ability to detect any symptom that can lead to a potential exacerbation. An exacerbation is defined as a worsening of a patient's symptoms from his or her usual stable state. The symptoms can then be analyzed by smart algorithms or health care professionals to detect the potential risk of an exacerbation [15]. It is still debatable whether telemonitoring on its own can help reduce hospitalization. Telemonitoring of patients’ condition, behavior (ie, physical activity, adherence to medication) and symptoms may be assistive in the early detection and treatment of an exacerbation of COPD, and in turn improve patients’ quality of life and reduce the high costs associated with COPD exacerbations The Telescot program investigated the impact of a telemetric COPD monitoring service. Results did not show any reduction in hospital admissions or improvement in quality of life [12]. However, there have been some tools such as TEXAS and EXACT to quantify and measure exacerbations in patients with COPD patients [15,16]. The validity of TEXAS, an automated telephonic exacerbation assessment system which records symptoms and use of medication, has been assessed. The study was carried out on 86 patients with COPD. The results showed that TEXAS, when compared with other tools such as a paper diary or medical record review, showed the highest detected rates of exacerbations and patients’ compliance in providing exacerbation-related information [16]. The EXACT daily diary was designed to standardize the process for assessing acute exacerbations of patients with COPD by providing a direct measure of patient-reported symptoms of exacerbation [15]. It is the first instrument that went through a qualification review by the Food and Drug Administration (FDA). Thus, systems like TEXAS and EXACT, if integrated into existing clinical services, can be helpful in providing the necessary information to help detect any potential exacerbations in patients with COPD and help improve their condition.

Another important feature for a COPD support tool is the self-management of physical activity. Improvement of physical activity levels could not only result in better physical functioning and less dyspnea, but also in a higher quality of life and lower risks for exacerbation-related hospitalization and mortality. The COPD Self-Management Activation Research Trial (SMART action) was designed for patients with COPD who were unable to attend a pulmonary rehabilitation center. Patients were receiving COPD self-management education by a health coach and weekly telephone calls for monitoring purposes [17]. Having a mHealth system that could be used to follow up patient’s physical activities would be beneficial to both health care providers and patients.

A recent manual, Self-Management Program of Activity, Coping and Education (SPACE), was developed in 2012 which included a self-report chronic respiratory questionnaire, an incremental shuttle-walk test, and an endurance shuttle walking test for patients with COPD [18]. Patients were observed during at the 6-week and 6-month follow-ups. The pilot study showed significant improvement for dyspnea management, exercise capacity, and breathlessness. SPACE also showed improvement in quality of life, endurance capacity, and reduced depression. The SPACE manual focused on education and behavior change for successful self-management of COPD. Such a manual could potentially be incorporated in a mHealth system to allow patients to refer to the self-management program remotely.

In the Netherlands, an autonomous mobile system for the management of COPD was piloted with actual patients with COPD to see the effectiveness of such a system in detecting and preventing exacerbations [19]. The main components of the COPD management system include a mobile phone, an intelligent model that works out algorithms based on the patient’s input, and a Web Center that reports the health conditions of patients to physicians. The mobile phone app, known as “AERIAL” has many identified features essential for COPD management, for example, patients have to fill questionnaires regularly for follow-up and send pulse-oximeter and micro-spirometer readings via Bluetooth to a central server to be monitored by health care professionals. The app was developed on the Android platform. To improve the system in terms of performance, error logs, and phone interface, it was evaluated with five patients and the results obtained from the first evaluation of two patients. Patients found the system easy to use but further research has to be carried out to see whether the system is accurate enough to predict exacerbations based on patient’s data. Overall the study showed that there is potential in improving the quality of life of patients using similar systems; however, the system did not include an educational program about medication, inhalation techniques, or smoking cessation [19].

Other COPD management systems deployed on mobile apps (ie, Me&MyCOPD and CGI CommunityCare360) are mHealth systems that allow patients to access personalized coaching and real-time information about their disease and treatment. Me&MyCOPD also allows patients to collect, transmit, and access their clinical data [20]. Health care professionals are able to monitor medication adherence and other features such as device tracking, patient training, managing clinic visits, and providing advice on lifestyle management. The mobile app CGI CommunityCare360 Health was developed by the Canadian company CGI Group and makes use of integrated care to connect patients, primary care physicians, case coordinators, work coordinators, extended care teams, mobile care providers, first responders, administrators, and managers [21]. It improves care coordination and patient empowerment. It also supports patients suffering from chronic diseases through questionnaires and a telemonitoring system to communicate with the health care providers directly through email messaging.

Effing et al [22] identified the following components of a self-management program for patients with COPD: (1) smoking cessation advice and support, (2) self-recognition and treatment of exacerbations, (3) exercise and increased physical activities, (4) nutritional advice, and (5) dyspnea management. Self-registration techniques such as diaries or tools to measure variables are other features identified to be useful for patients with COPD [23]. The study emphasized that since patients with COPD are mostly aged above 50 years old, apps developed for them need to include a user-centered design appropriate for use by the elderly.

Another paper showed that there is a need for personalized feedback for chronic disease management systems [24]. The study by Chomutare et al highlighted some of the missing links such as personalized education and core features (eg, a social network) that could have an impact on clinical outcomes.

The M-COPD system was developed by the Australian E-Health Research Centre, CSIRO (AEHRC) to enrich the link between patients and health care professionals. The system consists of a Web portal for clinicians and a mobile app for the self-assessment of symptoms and vital signs such as sputum, wheezing, cough, heart rate, and body temperature [25]. The main features identified were (1) reports; (2) email and mobile short message service (SMS); (3) monitoring; (4) reminders; and (5) education. The inconvenience for patients in such systems is that they need to use devices on their own at home to record body temperature, heart rate and pulse oximetry, and upload their measured values through the mobile phone.

In a clinical trial at the Royal Perth Hospital in Western Australia, 10 patients with COPD were recruited for a 3-month period to evaluate the M-COPD system [25]. Patients had to manually input observed and measured clinical data via a mobile Web browser. Results showed that the M-COPD system was very useful in terms of delivering patients’ data to clinicians in real-time so that the latter can remotely assist patients and deliver the right intervention. M-COPD was also found to be cost-effective as it results in large savings of time and costs compared to traditional nurse-visit programs. Overall, the M-COPD system showed great potential in improving the treatment and diagnosis of exacerbations and could also be used to track other symptoms and alert nurses in case symptoms moved above an assigned threshold.

Leading a healthy lifestyle can enhance the health of patients with COPD and reduce hospitalizations. Beattie et al [13] investigated how technology could be used to support lifestyle through a self-management app called CALS: COPD Lifestyle Support through Self-Management. According to this study, features that are necessary for a lifestyle support tool are (1) a smoking cessation program; (2) medication adherence; (3) healthy diet; (4) exercise; (5) breathing techniques; and (6) education. The study also highlighted that another important feature is the use of behavioral change and self-efficacy through the delivery of educational content, goal setting, related feedback, and monitoring of symptomatic features. By using such psychological factors, it can be determined whether a patient with COPD can perform or should avoid certain activities [26] and how to encourage patients to follow a specific program. Human behavior understanding (HBU) has been applied in various systems to support COPD (eg, monitoring a patient, medication intake, status monitoring, and daily activities) [27]. It must be noted that such behavioral support features were not evident in any of the 20 apps identified in this study.

The health of patients with COPD is related to environmental factors such as (1) temperature; (2) pollution level; (3) humidity; and (4) the chemical composition of air. COPD24 is a system that is based on telemonitoring and tele-treatment for patients with COPD. The COPD24 project takes into account the monitoring of patients’ vital signs as well as the surrounding environment, via dedicated sensors deployed within a wearable body area network (BAN) system and other meteorological sensors. Transmission of vital signs is achieved via wireless connections to health care providers and feedback is sent to patients in real-time. Air quality information is provided by the COPD24 service to warn patients about any hazardous areas that could affect their health. This system was evaluated with 30 patients over a 3-months trial period and showed that it is very important to make the patients self-manage their condition by monitoring both their symptoms and environment [28].

### Features Identified in Apps

After the first selection process, the 48 apps identified were listed in a spreadsheet and 29 (60%, 29/48) were categorized as medical, 17 (35%, 17/48) as educational, and 2 (4%, 2/48) as social network apps. Of those, 20 apps were downloaded on Android (70%, 14/20) and iOS (30%, 6/20) mobile devices and studied to identify some common features in those applications (Table 1).

**Table 1:** List of mobile apps and their features.

Name	Type^a^	Features
Chronic Obstructive Pulmonary Disease (COPD) @Point of Care	Medical	My Treatment, My Lab Results, My Journal, My Exacerbations, My Side Effects, My Medical History, patient education, charts
COPD^b^	Educational	Medical history, spirometry, medical examination, education, calendar
Pulmonology pocket	Educational	Guidance, assessment, monitoring, interactive calculators, index search, medication table
Miniatlas COPD	Medical	Education, communication, images
COPD (Chronic Obstructive Pulmonary Disease) Guide	Educational	Education
COPDexchange	Medical	Education, calculators
COPD Diary Card	Medical	Diary, mailing
Me&MyCOPD	Medical	Goals, status, advice, care plan, education
Calculate by QxMD	Medical/Calculator	Medical Calculators
Daxas – HCP	Educational	Guides, education for health care providers
Pulm- Pulmonology Pocket	Educational	Education, calculators, treatment guidelines, medication table
Pranayama Free	Educational	Breathing techniques (education)
COPD Guide	Educational	Education, guidelines
COPD Guide	Educational	N/A
PulmCCM	Educational	Education, guidelines
MEDGuide Emergency	Medical	Quick reference for emergencies, use of drugs (education)
Breathefree App	Medical	Choosing inhalers (education), calendar, medical news, severity of COPD
CGI CC360 HealthCenter Phone	Medical	Overview, results and goals, diary, questionnaire, calendar, device result, My Medication
ConnectMyCare	Medical	My Nurse, My symptoms, My Journal, My Appointments, My Medications, My Resources, My Providers, My Questions, reminders, calendar
palmEM: Emergency Medicine	Medical	Quick medication reference (education)

^a^Type includes medical, educational, or social.

^b^COPD: chronic obstructive pulmonary disease.

Many of the downloaded apps showed similar functionalities such as tracking of symptoms, exacerbations, questionnaires, and educational material. Out of the 20 identified COPD apps, 11 (55%, 11/20) were categorized as medical, 9 (45%, 9/20) as educational, and none as social network apps. Medical apps provide contact with health care professionals, lifestyle management applications, symptom tracking, or a list of medication. Educational apps are those which only provide guidance on COPD management to both patients and health care professionals to be used as a reference; they do not take any input from patients. Many such apps enhance the understanding of disease management and provide useful videos and links to other COPD forums. It has been observed that the number of medical COPD apps found across different platforms exceeded the number of educational or social networking apps.

The mobile apps that we downloaded had many features in common. A guide to the treatment and management of COPD and educational resources including videos, forums, and information including therapies and oxygen therapy were found to be the most common. Some apps had assessment scales and lookup tables related to COPD. Tools such as calculators for spirometry, body mass index (BMI), or tobacco consumption were also included in some of the apps to monitor patients’ lifestyle factors that could affect their condition. A management tool to enable patients to track and store relevant health information between clinician visits was also a feature in many of the downloaded apps. This included diaries related to (1) food and nutrition; (2) medications; (3) symptoms; (4) measurements; (5) physical activity; and (6) sleep. Other functionalities present were email messaging or any type of communication with health care providers. Finally, a tracking symptom feature in order to see if there is any improvement or deterioration in patients’ health condition was also included in some apps. The most common features and the percentages of the selected apps containing those features are shown in Table 2.

**Table 2:** The number of downloaded apps containing the identified common features (N=20).

Feature	n (%)
Education	13 (65)
Medication/ treatment	5 (25)
Guidelines	5 (25)
Look-up tables	3 (15)
Symptom tracking	3 (15)
Diary or calendar	6 (30)
History	2 (10)
Email	6 (30)
Calculators	4 (20)
Others	5 (25)

## Discussion

### Principal Findings

The findings presented here provide new insights into the potential features that should be considered in designing a mHealth system to assist patients suffering from COPD. They highlight not only the necessary essential tools but also the programs needed to support patients with COPD [13]. When designing systems for self-management of chronic diseases, we must consider other factors such as age, information technology experience, education level [12], and possible comorbidities [12,14]. Moreover, all educational material has to be retrieved from a trustworthy source such as the National Institute for Health and Care Excellence (NICE) or Global Initiative for Chronic Obstructive Lung Disease (GOLD) guidelines. The platform tool can also be used to boost patient’s psychological motivation and help them adhere to medication by using different features like questionnaires or diaries (Table 2).

**Figure 3:**
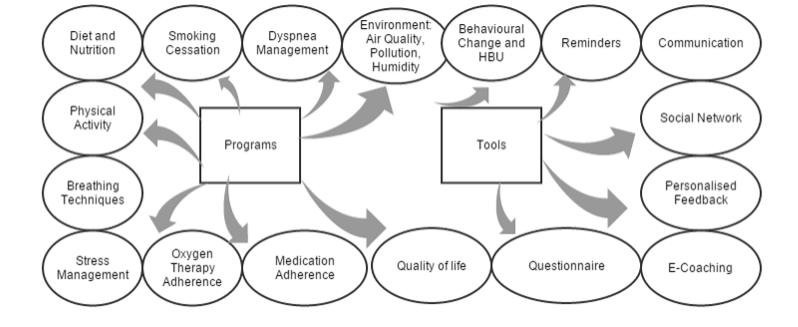
Features, including tools and programs, identified in this study that should be taken into consideration when building a support tool for patients with chronic obstructive pulmonary disease.

Mobile technologies and telehealth have the potential to provide patients with COPD a better quality of life if the vital features are incorporated in an app. An important parameter is that every COPD exacerbation event has a gradual increment phase preceding the peak exacerbation time for up to several days and a potential early detection of such a tendency towards this peak event could prevent its occurrence and lead to a significantly milder clinical presentation. Therefore, the use of a system for the early diagnosis of evolving exacerbations is expected to be very cost-effective and could diminish the cost of severe deteriorations [29]. There may also be reductions in health complications and hospital admissions, but this is still debatable [12,25,30,31]. Although there is little published material on the effectiveness of the identified COPD apps, previous studies identified the desired features for a COPD support app. The features for a support tool for a patient app are summarized in Figure 3. So far, the apps identified on the market are limited in terms of functionalities and very few of them emphasize the needs of patients with COPD with comorbidities.

Other features (ie, social networking tools) can be important as they allow patients to share information about their personal experience, symptoms, treatments, and outcomes. Some apps allow patients suffering from different diseases to share data and discuss their health with health care professionals, thus improving knowledge sharing [32]. Through collaborating and knowledge, patients from diverse clinical backgrounds may feel better and knowledge-based communities can be formed [33]. Missing links such as personalized information, education about COPD, and electronic coaching (e-coaching) features in an app could improve the way patients manage their lifestyle.

Since COPD is a highly symptomatic disease, patients may not recognize small day-to-day variations in their pulmonary symptoms. Lack of symptom awareness and pace of symptom worsening make daily telemonitoring of patients with COPD an attractive and beneficial approach to facilitate an early intervention. Telehealth also has the potential to allow health care professionals to monitor patients remotely for deteriorations or long-term trends and offers opportunities for intervention to improve outcomes. They can view the data of their patients on a constant basis, not only periodically at the outpatient clinics. They are also able to define the current health status of their patients and provide coaching on how to cope with certain adverse symptoms or receive the proper treatment. In addition, they can send motivational messages to patients in order to ensure that they perform their regular exercises, follow the assigned smoking cessation program, and so on. Moreover, telemedicine may be beneficial for obtaining an active lifestyle by increasing patients’ awareness through self-monitoring, goal setting, and improving self-efficacy. On the other hand, a recent review reported concerns from health care professionals that telehealth may promote patients’ dependency on health care providers and telehealth data, especially in the more severe patients. In addition, health care professionals indicated that the technical type of work brought by telehealth increases burden and undermines aspects of their professional identity [34].

**Figure 4:**
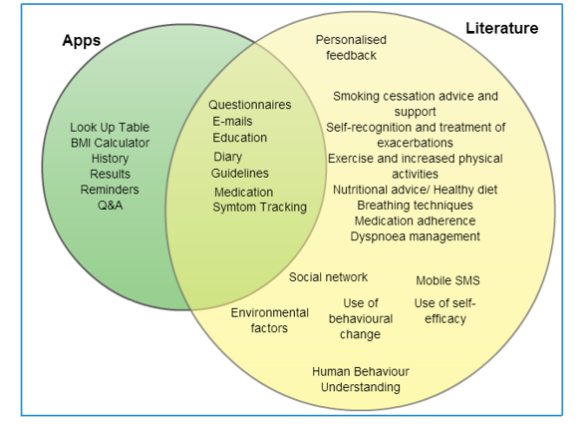
Common features identified in the downladed apps and in the literature.

Our study has shown that the majority of the identified apps had an education section, whereas some of them referred to medication and guidelines, and about one third included a calendar or a diary and many other features such as reminders or symptom tracking. This literature and pilot study describe the different features for which patients with COPD should be monitored by mobile apps (Figure 3). The common features identified from the downloaded apps and from the literature are shown in Figure 4.

### Limitations

There are many apps available in the various online markets; after limiting the search results, 214 apps were identified. However, due to limited resources, only 20 apps were downloaded. More apps could be downloaded to review their features for further research. Moreover, only native mobile apps were searched; no mobile Web-based apps were considered.

### Conclusions

The management of COPD requires a multidisciplinary approach involving many different types of treatment; currently the system is very segregated. While many of the features identified such as questionnaires, emails, education, or diary tools were found to be common in this study, others like social networking tools, personalized education, feedback, e-coaching, and psychological motivation to enhance behavioral change have been found to be missing in many studies and apps. This shows that not enough research has been conducted to analyze features for a COPD support tool.

Many features seem to have been considered in the literature but are not implemented in current support apps targeting patients with COPD with different comorbidities. Hence, these features can and should be incorporated in a single app for better monitoring, follow-up by health care professionals, and lifestyle management for patients with COPD. This can lead to a balance between obtaining improved clinical outcomes with minimal inconvenience to the patient. The tool can be mainly designed for elderly patients with a user friendly interface to collect data which will be easily accessible to health care professionals. The design of the proposed app must be followed by an evaluation of such a self-management support tool to study its impact on patients’ health outcome as the literature is scarce in this regard.

## Abbreviations

COPD: chronic obstructive pulmonary disease
e-coaching: electronic coaching
mHealth: mobile health
SMART: Self-Management Activation Research Trial
SPACE: Self-Management Programme of Activity, Coping and Education
